# Supplementary figures and images for: Knockdown of the translocon protein EXP2 in Plasmodium falciparum reduces growth and protein export
Source: PLoS One. 2018 Nov 15;13(11):e0204785. doi: 10.1371/journal.pone.0204785 (PMC6237293; doi:10.1371/journal.pone.0204785)

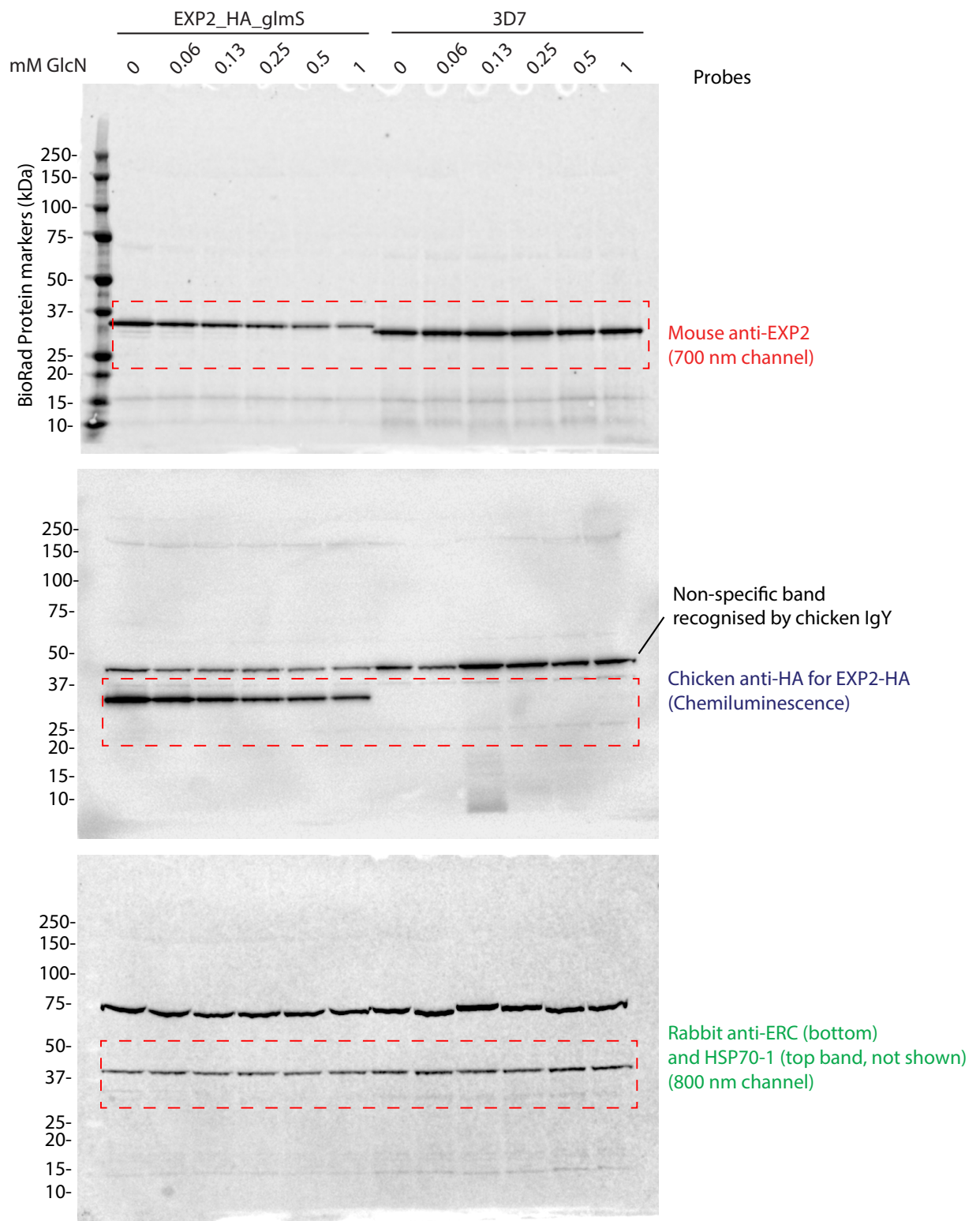

Supplement: S1 Fig — (PDF) [file pone.0204785.s001.pdf]
