# Supplementary material for: Knockdown of the translocon protein EXP2 in Plasmodium falciparum reduces growth and protein export
Source: PLoS One. 2018 Nov 15;13(11):e0204785. doi: 10.1371/journal.pone.0204785 (PMC6237293; doi:10.1371/journal.pone.0204785)
Supplement: S2 Fig — (PDF) [file pone.0204785.s002.pdf]

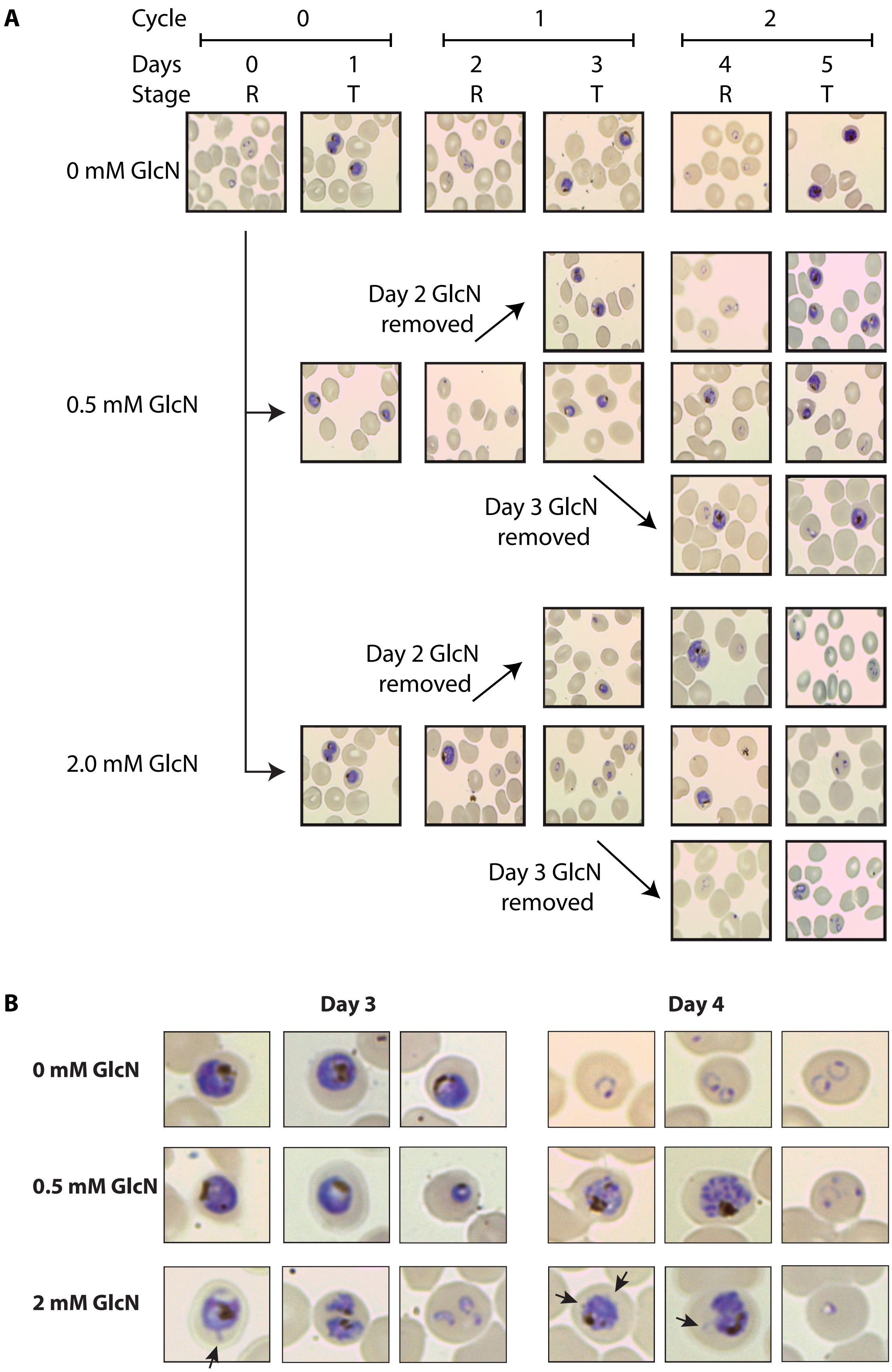

S2 Fig. Knockdown of EXP2-HAglmS inhibits parasite growth. A. Giemsa stained thin blood smears showing growth slowed growth and parasite death caused by knockdown of EXP2-HAglmS. Parasites smears are from experiment show in Fig 3. **B.** Magnified parasite images from Day 3 and 4 with arrows showing surface protrusions caused by knockdown of EXP2-HA.
