# Supplementary material for: Knockdown of the translocon protein EXP2 in Plasmodium falciparum reduces growth and protein export
Source: PLoS One. 2018 Nov 15;13(11):e0204785. doi: 10.1371/journal.pone.0204785 (PMC6237293; doi:10.1371/journal.pone.0204785)
Supplement: S3 Fig — (PDF) [file pone.0204785.s003.pdf]

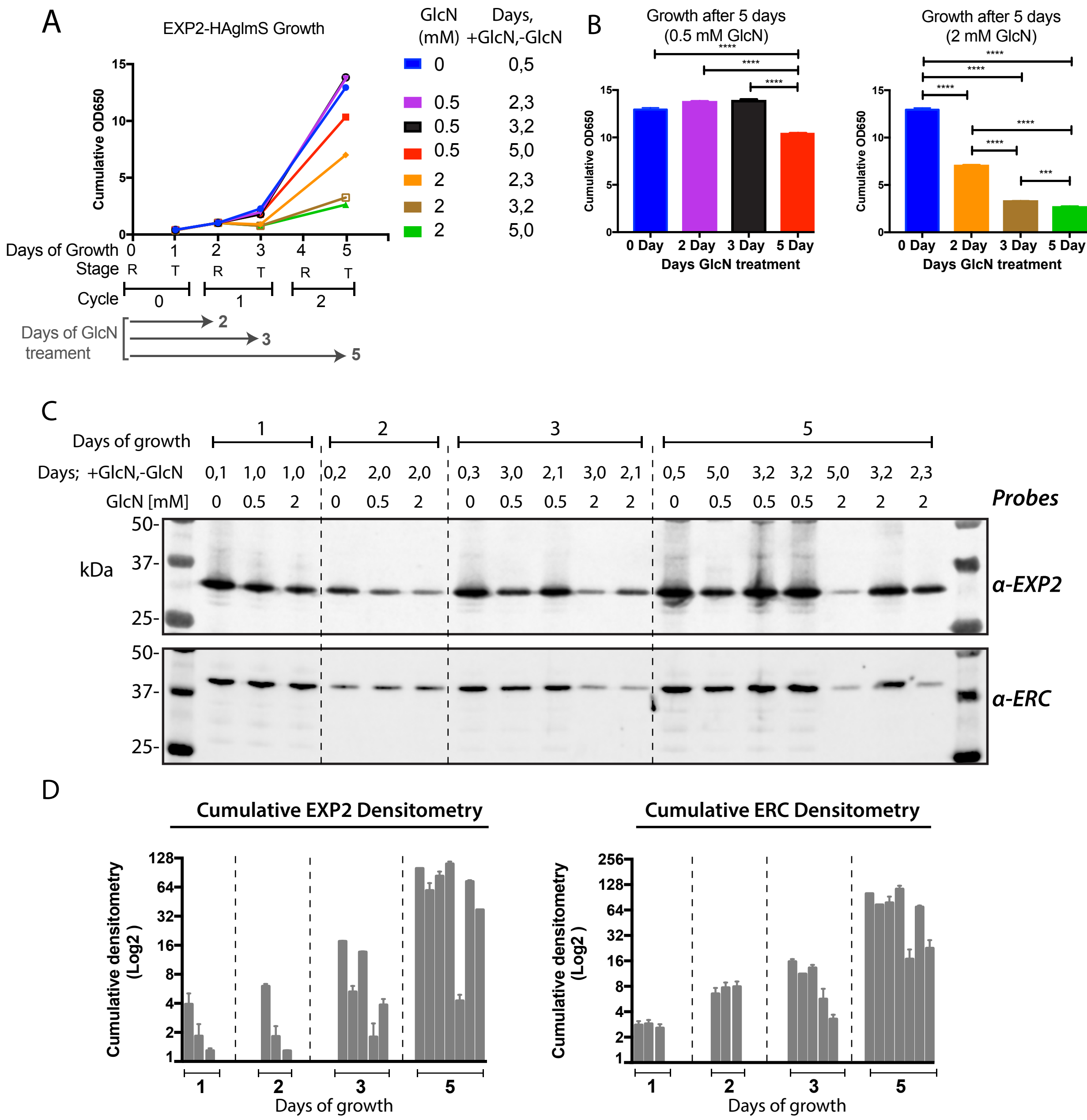

S3 Fig. After EXP2-HA is knocked down parasite restoration of parasite growth is dependent upon EXP2 expression. (A) GlcN was added to 3% ring stage EXP2-HAglmS parasites to 0, 0.5 and 2 mM on day 0, cell cycle 0. To prevent the parasites from over-growing those that were to be harvested at cycle 1 and 2 were diluted 1/5 and 1/25, respectively at the start of the assay. The parasites were grown for 5 days (2 cell cycles) with samples removed for growth and western blot analysis on day 1 (cycle 0), day 2 and day 3 (cycle 1) and day 5 (cycle 2). To assess recovery of growth after EXP2 knockdown, some of the parasites were only treated with GlcN for 2 or 3 days after which the GlcN was removed and growth continued until day 5. To quantify growth, parasite lactate dehydrogenase (LDH) activity of 3 technical replicates was measured on each day. The LDH values (OD 650 nm) were multiplied by the parasite dilution factor to produce a cumulative measure of growth. **(B)** Growth at day 5 after continuous GlcN treatment compared to 2 and 3 days GlcN treament and recovery until day 5. Cumulative OD650 values taken from **(A)**, indicate that growth recovery is higher the shorter the GlcN treatment period and the lower the concentration used. Tukey's multiple comparison,*** $p<0.001$, ****$p<0.0001$. **(C,D)** Western blot analysis and cumulative densitometry of parasite samples taken each day as indicated in **(A)**, shows that EXP2 knockdown is proportional to the GlcN concentration used and the degree of growth inhibition. The loading control ERC, is proportional to parasite biomass indicated by LDH activity and tends to trail behind the degree of EXP2 knockdown. One representative western blot of two is shown. The graph bars indicate cumulative densitometry of the western blots bands (densitometry x dilution factor) and show mean ± range of n=2. The graph bars have the same order and identity as the western blot bands in **(C)** .
